# Maternal Thyroid Function During Pregnancy and Offspring White Matter Microstructure in Early Adulthood: A Prospective Birth Cohort Study

**DOI:** 10.1089/thy.2022.0699

**Published:** 2023-10-13

**Authors:** Lassi Björnholm, Olavi Orell, Martta Kerkelä, Ulriika Honka, Sini Laasonen, Tiina Riekki, Heljä-Marja Surcel, Eila Suvanto, Juha Veijola

**Affiliations:** ^1^ Research Unit of Clinical Medicine, Department of Psychiatry, University of Oulu, Oulu, Finland.; ^2^ Department of Psychiatry, Oulu University Hospital, Oulu, Finland.; ^3^ Medical Research Centre Oulu, Oulu University Hospital and University of Oulu, Oulu, Finland.; ^4^ Research Unit of Health Sciences and Technology, Faculty of Medicine, University of Oulu, Oulu, Finland.; ^5^ Institute of Biomedicine, University of Eastern Finland, Kuopio, Finland.; ^6^ Faculty of Medicine, University of Oulu, Oulu, Finland.; ^7^ Department of Obstetrics and Gynecology, Oulu University Hospital and MRC Oulu University, Oulu, Finland.

**Keywords:** thyroid, hypothyroidism, pregnancy, early adulthood, white matter, axon

## Abstract

**Background:**

The fetus is fully dependent on maternal thyroid hormones until mid-gestation and suboptimal maternal thyroid function has been associated with alterations in the neurodevelopment of the offspring. We used maternal free thyroxine (fT4) and thyrotropin (TSH) levels in early gestation to study the association of maternal thyroid function during early pregnancy and offspring brain white matter (WM) integrity in early adulthood.

**Methods:**

Our study population consisted of a total of 292 mother–child pairs. Maternal fT4 and TSH were used as predictors and offspring multimodal imaging measures of fractional anisotropy, mean diffusivity, and magnetization transfer ratio (FA, MD, and MTR) as dependent variables. First, as Global analysis, all analyzed 14 WM tracts were studied simultaneously using linear-mixed effect models. Second, if a global effect was detected, a *post hoc* Tract-wise analysis was carried out using linear models individually in each WM tract. Study population was stratified by sex.

**Results:**

We found a positive association between maternal fT4 and offspring Global FA in males when adjusted for all maternal and offspring covariates (*n* = 114; β = 0.154; confidence interval = 0.045–0.263; *p* = 0.006). The finding was observed to be driven by multiple WM tracts, of which three projection fiber tracts and the forceps minor survived correcting for multiple comparisons in Tract-wise analysis.

**Conclusions:**

Maternal thyroid function in early pregnancy was observed to be associated with WM microstructure in male offspring in early adulthood. Our results suggest that maternal fT4 levels in early pregnancy may modulate axonal characteristics, with a long-term effect on offspring WM development.

## Introduction

The fetus is fully dependent on maternal thyroid hormones (THs) until the gradual activation of fetal hormone production from mid-gestation onward.^1^ Subclinical hypo- and hyperthyroidism are relatively common among pregnant women, with prevalence of 3.47% and 2.18%, respectively, during the first trimester.^2–4^ Clinically marked hypothyroidism may cause severe defects in growth and neurological development of the offspring and, most severely, neurological cretinism.^5^

Maternal hypothyroidism has been shown to affect offspring growth, for instance, by reducing birth weight and head circumference in neonates.^6^ Maternal thyroid deficiency has been associated with lower mean intelligence quotient (IQ)^7,8^ and intellectual disability of the offspring.^9^ Furthermore, low maternal free thyroxine (fT4) concentration during early pregnancy has been associated with increased risk of offspring psychomotor^10^ and mental developmental impairment,^8^ including increased risk of schizophrenia.^11^ Maternal thyroid function in early gestation has been also associated with higher prevalence of offspring attention-deficit and hyperactivity disorder (ADHD) symptoms (positive relation to maternal thyrotropin [TSH])^12^ and poorer scholastic performance (positive relation to maternal subclinical hypothyroidism or hyperthyroidism)^13^ in the offspring.

The effects on cognition may not be preventable using levothyroxine treatment of hypothyroid mothers at early or mid-pregnancy.^14,15^ Optimal maternal thyroid function during pregnancy seems to follow an inverted U-shaped profile, as also maternal hyperthyroidism during the first trimester has been linked with lower mean IQ^16^ and autism spectrum symptoms^17,18^ in the offspring. Based on earlier findings, it is likely that suboptimal maternal thyroid function in early pregnancy impacts long-term neurodevelopmental outcomes in the offspring.

THs are critically involved in the development of the central nervous system (CNS) through specific time windows influencing neurogenesis, neuronal migration, neuronal and glial cell differentiation, myelination, and synaptogenesis.^19^ Animal studies have reported irreversible offspring brain developmental outcomes in moderate and transient deficiency of THs in early gestation, stressing the neurodevelopmental impact of even mild thyroid dysfunction during the first trimester.^20–23^ Human neuroimaging studies on maternal thyroid function and offspring brain outcomes are, however, scarce. A large study in a prospective birth cohort recently reported an inverted U-shaped association between maternal TSH and offspring total gray matter (GM) and cortical GM volumes, independent of total brain volume, in childhood.^24^ In addition, associations between maternal hypothyroidism and variation in partial volumes and shape of the corpus callosum (CC),^25^ as well as reduced hippocampal volumes,^26^ have been reported in childhood, although these two studies were restricted by small sample size.

White matter (WM) occupies about half of the human brain, with axonal pathways transferring and modulating information across brain regions. The early organization of WM is set during the first and the second trimesters. Optimal early organization of WM circuits is crucial for cognitive development,^27^ but also highly vulnerable to disruptions in maternal TH function. In this study, we examine the association between maternal thyroid function in early pregnancy and offspring brain WM microstructure in young adulthood in a prospective birth cohort sample. Maternal TSH and fT4 are used to assess thyroid function. An explorative analysis of 14 major WM tracts is conducted using *in vivo* neuroimaging modalities of tissue microstructure: diffusion tensor imaging (DTI) metrics, fractional anisotropy (FA), and mean diffusivity (MD), and magnetization transfer ratio (MTR).

Earlier research suggests altered brain developmental trajectories in children of mothers with suboptimal gestational thyroid function but the relation in adulthood remains unexplored. WM development has been shown to follow considerably different trajectories in males and females.^28–31^ Furthermore, based on the sexual dimorphism of offspring development in relation to prenatal maternal thyroid function^12,13,32,33^ and the greater vulnerability of the male fetus to early developmental insults as implied by some studies,^33,34^ we decided to study males and females separately. Based on previous work in children and preclinical models, we hypothesize that prenatal maternal thyroid function is associated with sex-specific alterations in WM microstructure in the offspring in young adulthood.

## Materials and Methods

### Northern Finland Birth Cohort 1986

The Northern Finland Birth Cohort 1986 (NFBC1986, www.oulu.fi/nfbc) is a population-based prospective birth cohort consisting of all the expected pregnancies between July 1, 1985 and June 30, 1986 in the two northernmost provinces of Finland (Oulu and Lapland) covering 99% of pregnancies in 1 year (*n* = 9362). Data collection started at first prenatal care visit and continued with further follow-ups of the offspring at 7–8 and 15–16 years of age. A subsample of 26-year-olds was selected for magnetic resonance imaging (MRI) acquisition based on maternal smoking status during pregnancy. The MRI scanning was completed successfully on 451 participants.^28^ Exclusion criteria for the follow-up MRI are given in the Supplementary Methods. Maternal thyroid disorder or treatment of thyroid disorder was not included in the exclusion criteria.

The study protocol was approved by the ethics committee of Northern Ostrobothnia Hospital District (decision EETTMK:53/2011). Informed written consent was obtained from all subjects. Data of maternal thyroid function during pregnancy were obtained from the Finnish Maternity Cohort (FMC). The use of the samples was approved by the scientific committee of the Biobank Borealis of Northern Finland.

### Image acquisition and processing

Each individual's MRI data were collected during a single session on a GE Signa 1.5 T scanner. Processing of diffusion imaging data included corrections for imaging errors, after which semi-automated AutoPtx^35^ pipeline, which consists of FMRIB Software Library (FSL, https://fsl.fmrib.ox.ac.uk/fsl/fslwiki/FSL) tools, was used to run probabilistic tractography in participant native space to extract brainstem, projection, association, callosal, thalamic, and limbic system fibers, resulting in 27 WM tracts in each individual. Further details of image acquisition and processing are provided in Supplementary Methods.

### Sampling of thyroid markers during pregnancy

Data of maternal thyroid function were available for 5805 children (61.2% of the NFBC1986). As part of the FMC of >950,000 women, ∼1–3 mL of serum was collected from each participant during a routine screening for congenital infections at mean 11.0 (standard deviation [SD] 3.6) weeks of gestation. Informed consent was provided by the women and the samples were stored in a biorepository at −25°C at Biobank Borealis, Oulu, Finland. Serum samples were analyzed for TSH and fT4 using an Architect i2000 automatic analyzer.^13^ Reliable analysis of the thyroid markers is possible after 23 years of storage and the effects have been studied earlier.^36^

### Covariates

We chose potential covariates based on prior literature of maternal thyroid function and the child's neurodevelopment. We used child sex, gestational age at birth (full gestational weeks), age at image acquisition, and brain volume (estimated total intracranial volume, eTIV) as offspring covariates. Maternal covariates included age at childbirth, socioeconomic status, prepregnancy body mass index (BMI), cigarette smoking, and alcohol use during pregnancy. Maternal gestational thyroid peroxidase (TPO) antibody level was used as a covariate in Sensitivity analysis 2. Offspring IQ was calculated using vocabulary and matrix reasoning profiles and cigarette smoking and education were self-reported at 26 years neuroimaging follow-up. Maternal cigarette smoking was determined during a visit to antenatal clinic and was considered positive if the mother continued smoking one or more cigarettes per day in the second trimester of pregnancy.

Alcohol use during pregnancy was assessed in a questionnaire given to mothers. Self-reported mother's socioeconomic status was assessed using a scale of 1–5, 1 being upper higher income worker and 5 a stay-at-home mother.^37^

### Statistical analysis

The maternal thyroid and offspring MRI parameters were treated as continuous variables and outliers greater than ±3 SD were excluded. Of 27 WM fiber tracts, the left and right counterpart of 24 bilateral tracts were averaged pairwise, resulting in a total of 15 tracts. The *acoustic radiation* tract was excluded owing to tractography pipeline failing to capture the tract properly, leaving 14 WM tracts for the analysis (see Supplementary Methods).

All variables were standardized (z-score) by sex to produce comparable estimates of the unitless brain imaging measures. As a “Global analysis,” the associations between maternal thyroid markers (fT4 and TSH) and WM tracts were studied using linear mixed-effects (LME) models in each offspring neuroimaging measure (FA, MD, MTR). The LME model reduces the number of tests by considering all tracts in one model and is able to capture variation within subjects (intercept for each individual) and between subjects (intercept for each tract) and thus avoid averaging and reduction of data dimensionality. Random intercepts were given for participant and WM tract. Models were adjusted for offspring age at image acquisition (Model 1) and, additionally, maternal age at childbirth, socioeconomic status, prepregnancy BMI, cigarette smoking and alcohol use during pregnancy (Model 2), and, offspring gestational week at birth and brain volume at image acquisition (Model 3).

To study the contribution of individual tracts to any association observed in Global analysis, *post hoc* linear regressions fully adjusted for confounders (Model 3) were run for each tract of the given measure as “Tract-wise analysis.”

Data of males and females were also combined, standardized, and associations between maternal thyroid and offspring neuroimaging measures were studied using Global analysis and the three models. The associations were tested for interaction of sex in the fully adjusted model (Model 3) to test our hypothesis of sex differences in the main effect. Quadratic dependencies between maternal thyroid and offspring brain measures were studied by adding quadratic terms in LME (Model 3) in sex-stratified and combined analyses.

False discovery rate (FDR) with Benjamini–Hochberg method was used for adjusting *p*-values for multiple comparisons in Global analysis (*n*_FDR_ = 3) and in Tract-wise analysis (*n*_FDR_ = 14) and the resulting *q*-values <0.05 were considered significant. All statistical analyses were carried out using R Statistical Software version 1.1.456.^38^

As Sensitivity analysis 1, differences were studied in (1) maternal fT4 between offspring with and without brain imaging data and (2) in offspring brain volume (eTIV) between offspring with and without maternal thyroid function data using *t*-tests separately in males and females. As Sensitivity analysis 2, maternal TPO antibody level was used as a covariate in addition to the covariates used in Global analysis Model 3. As Sensitivity analysis 3, individuals with overt hypothyroidism (TSH >4 mU/L and fT4 < 11 pmol/L) and hyperthyroidism (TSH <0.5 mU/L and fT4 > 23 pmol/L) were excluded from analysis in Global analysis Model 3.

## Results

**FIG. 1.**
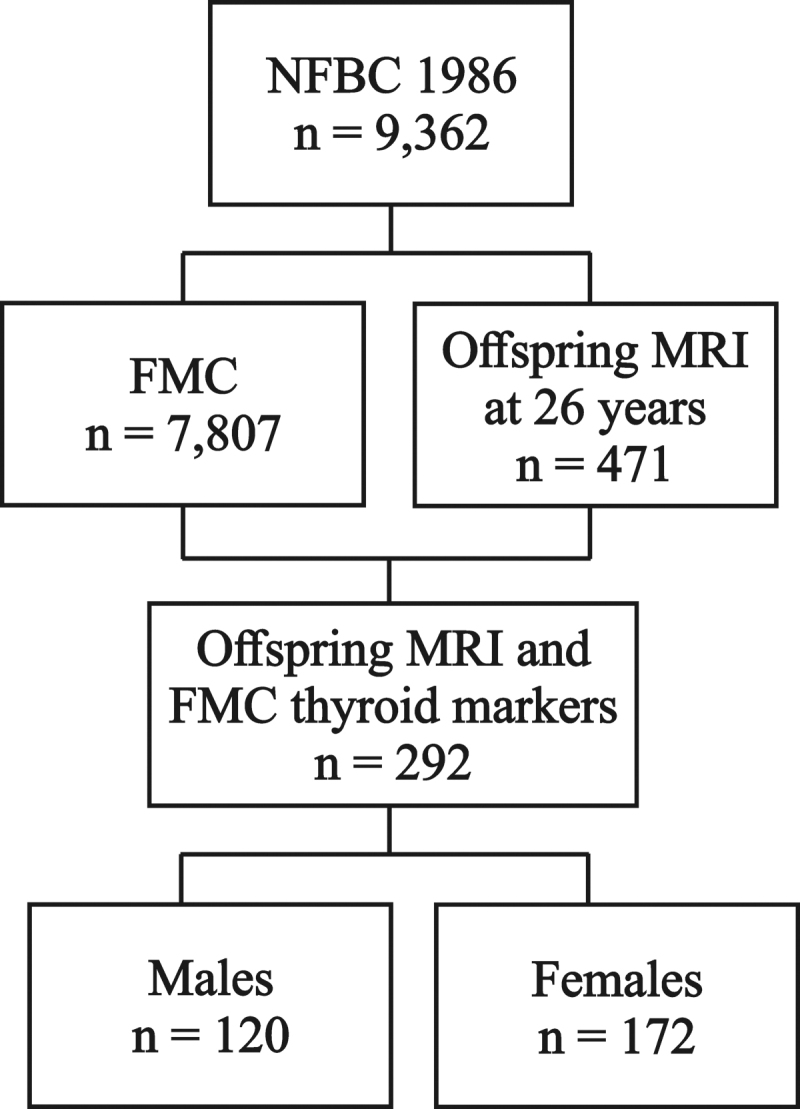
Flow diagram of the study population. FMC, Finnish Maternity Cohort; MRI, magnetic resonance imaging; NFBC1986, Northern Finland Birth Cohort 1986.

Of 7807 serum samples and 471 children with completed MRI, 299 mother–child pairs had usable maternal thyroid and offspring MRI data available. Two females were excluded due to outlier values in TSH (12.78 and 110 mU/L), one male and one female due to missing TSH and two males and one female due to missing fT4. In addition, one female was excluded from the FMC dataset due to outlier value in TSH (110 mU/L), only affecting Sensitivity analysis 1. The final study population consisted of 292 mother–child pairs with complete maternal thyroid and offspring MRI data (Fig. 1). The median (interquartile range) concentrations of fT4 (pmol/L) and TSH (mU/L) were 14.7 (2.1) and 1.1 (1.1) in male and 15.0 (2.8) and 1.1 (1.0) in female pregnancies (Table 1).

**Table 1.** Description of the Study Population

	Male,* N* = 120	Female,* N* = 172
Maternal variables		
Age (years, SD)	28.2 (5.2)	28.0 (5.2)
BMI (units, SD)	22.4 (3.3)	22.4 (4.4)
Data missing	4 (3.3%)	4 (2.3%)
Socioeconomic level		
Level 1	4 (3.3%)	3 (1.7%)
Level 2	13 (10.8%)	32 (18.6%)
Level 3	59 (49.2%)	68 (39.5%)
Level 4	36 (30.0%)	54 (31.4%)
Level 5	5 (4.2%)	12 (7.0%)
Data missing	3 (2.5%)	3 (1.7%)
Smoking during pregnancy		
Yes	59 (49.2%)	73 (42.4%)
No	61 (50.8%)	99 (57.6%)
Alcohol use during pregnancy		
Yes	14 (11.7%)	28 (16.3%)
No	106 (88.3%)	144 (83.7%)
Offspring variables		
Gestational age at birth (full weeks)	39.5 (1.3)	39.6 (1.4)
IQ	107.6 (18.9)	109.68 (20.3)
Education		
<9 Years of elementary school	3 (2.5%)	3 (1.7%)
Elementary school	62 (51.7%)	61 (35.5%)
Matriculation	59 (49.2%)	114 (66.3%)
Data missing	2 (1.7%)	3 (1.7%)
Cigarette smoking^a^	53 (44.2%)	57 (33.1%)
Age at MRI scan (years)	26.4 (0.5)	26.5 (0.5)
Brain volume (cm^3^)^b^	1731.8 (133.7)	1481 (145.4)
Exposure		
Maternal fT4 (pmol/L)	14.7 (2.1)	15.0 (2.8)
Maternal TSH (mU/L)	1.1 (1.1)	1.1 (1.0)
Gestational age at blood sampling (full weeks)	10.6 (4.8)	11.5 (5.0)
Data missing	2 (1.7%)	5 (2.9%)
Outcome		
FA ( × 100)	53.0 (7.3)	51.9 (7.7)
Data missing (single tract)	5 (4.2%)	7 (4.1%)
Data missing (multiple tracts)	0	1 (0.6%)
MD ( × 10,000)	7.8 (0.6)	7.8 (0.6)
Data missing (single tract)	4 (3.3%)	14 (8.1)
Data missing (multiple tracts)	0	1 (0.6%)
MTR	38.9 (1.5)	38.6 (1.5)
Data missing (single tract)	4 (3.3%)	11 (6.4%)
Data missing (multiple tracts)	0	0

Categorical variables are reported as number of participants (%) and continuous variables as mean of the participants (SD), maternal thyroid measures are reported using median and interquartile range. No data were missing for a given variable when left unreported.

^a^ Ever smoked cigarettes at least occasionally (try not counted).

^b^ Estimated total intracranial volume from Freesurfer.

BMI, body mass index; FA, fractional anisotropy; fT4, free thyroxine; IQ, intelligence quotient; MD, mean diffusivity; MRI, magnetic resonance imaging; MTR, magnetization transfer ratio; SD, standard deviation; TSH, thyrotropin.

Combined analyses of males and females did not show any linear or quadratic associations between maternal thyroid function and offspring microstructural measures (Supplementary Table S1). Interaction of sex was significant after correcting for multiple comparisons in maternal TSH and offspring FA (β = 0.179; confidence interval [CI] = 0.049–0.309) and MD (β = −0.158; CI = −0.291 to −0.026), but not TSH and MTR (β = 0.098; CI = −0.054 to 0.249) nor fT4 and the microstructural measures (FA: β = −0.134; CI = −0.272 to 0.005; MD: β = 0.037; CI = −0.104 to 0.178; MTR: β = 0.019; CI = −0.142 to 0.181).

**FIG. 2.**
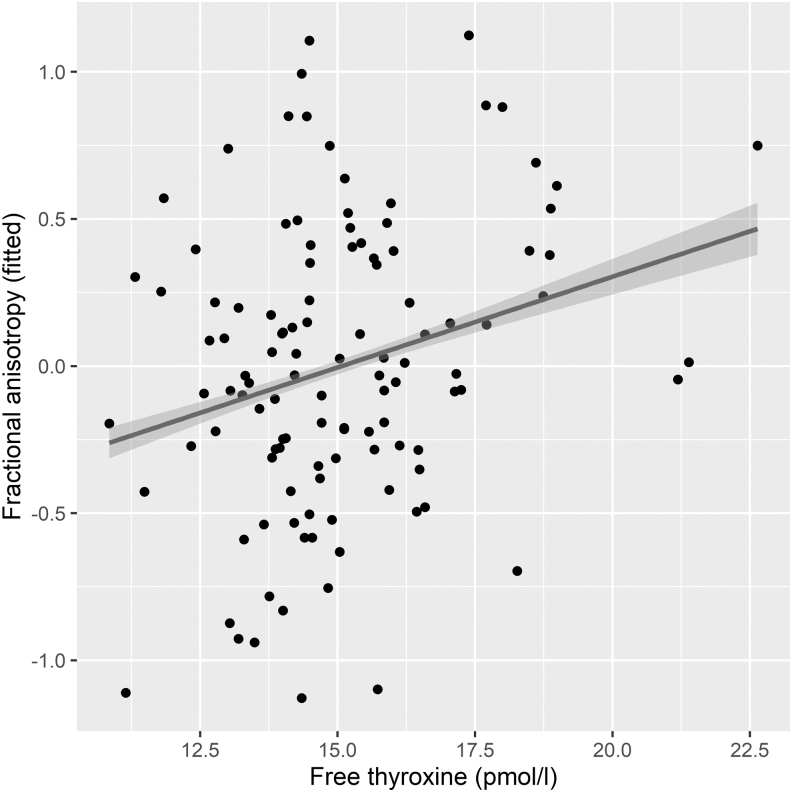
The association between maternal fT4 and offspring FA in males in Global analysis (all tracts) adjusted as in Model 3. FA, fractional anisotropy; fT4, free thyroxine.

We observed a positive association between maternal fT4 and Global FA in male offspring in the adjusted Models 2 and 3 (a in Table 2 and Fig. 2). In females, positive association was observed between maternal TSH and offspring Global FA (Model 2), but the association was lost after correcting for multiple comparisons in Model 3 (b in Table 2). The negative associations between TSH and MD (Models 2 and 3) in females did not survive correcting for multiple comparisons. No associations were observed between TSH and neuroimaging measures in males or fT4 and neuroimaging measures in females. No quadratic dependencies were observed between maternal thyroid and offspring neuroimaging measures in sex-stratified analyses.

**Table 2.** Associations Between Maternal Thyroid Hormones and Offspring Global Brain Microstructural Measures

(a) Males						
	Model 1 (*n* = 120)		Model 2 (*n* = 114)		Model 3 (*n* = 114)	
Global WM measures	β [CI]	*p*	β [CI]	*p*	β [CI]	*p*
fT4						
FA	0.147 [0.035 to 0.259]	0.011	0.163 [0.049 to 0.276]	0.006^a^	0.154 [0.045 to 0.263]	0.006^a^
MD	−0.056 [−0.168 to 0.055]	0.323	−0.088 [−0.202 to 0.026]	0.131	−0.092 [−0.206 to 0.021]	0.113
MTR	−0.019 [−0.149 to 0.111]	0.774	−0.007 [−0.140 to 0.127]	0.924	−0.025 [−0.150 to 0.099]	0.692
TSH						
FA	−0.040 [−0.138 to 0.058]	0.429	−0.045 [−0.145 to 0.054]	0.369	−0.053 [−0.148 to 0.041]	0.272
MD	0.031 [−0.065 to 0.126]	0.530	0.038 [−0.059 to 0.135]	0.446	0.038 [−0.059 to 0.135]	0.446
MTR	0.009 [−0.102 to 0.121]	0.871	0.014 [−0.099 to 0.127)]	0.808	0.011 [−0.095 to 0.117]	0.837

(b) Females						
	Model 1 (*n* = 172)		Model 2 (*n* = 165)		Model 3 (*n* = 165)	
Global WM measures	β [CI]	*p*	β [CI]	*p*	β [CI]	*p*
fT4						
FA	0.010 [−0.074 to 0.095]	0.813	0.003 [−0.082 to 0.087]	0.950	0.013 [−0.072 to 0.097]	0.767
MD	−0.063 [−0.146 to 0.019]	0.135	−0.050 [−0.132 to 0.032]	0.233	−0.049 [−0.132 to 0.034]	0.247
MTR	−0.003 [−0.102 to 0.096]	0.948	−0.010 [−0.110 to 0.090]	0.844	0.000 [−0.099 to 0.100]	0.993
TSH						
FA	0.118 [0.027 to 0.210]	0.012	0.128 [0.035 to 0.222]	0.008^a^	0.123 [0.029 to 0.217]	0.011
MD	−0.080 [−0.171 to 0.011]	0.088	−0.106 [−0.198 to −0.014]	0.026	−0.113 [−0.206 to −0.021]	0.017
MTR	0.101 [−0.007 to 0.208]	0.068	0.113 [0.001 to 0.224]	0.049	0.108 [−0.004 to 0.219]	0.059

Linear mixed-effects models were used in the analysis. β represents the association between fT4 or TSH and neuroimaging measures FA, MD, and MTR in all 14 WM tracts with CIs and raw *p*-values (*p*). Model 1 is adjusted for offspring sex and age at image acquisition, Model 2 additionally for maternal age, prepregnancy BMI, socioeconomic status, cigarette smoking, and alcohol use during pregnancy, and Model 3 additionally for offspring full gestational weeks at birth and brain size at early adulthood image acquisition. Number of participants available for each model is given in parenthesis.

^a^ *q* < 0.05.

CI, confidence interval; WM, white matter.

**FIG. 3.**
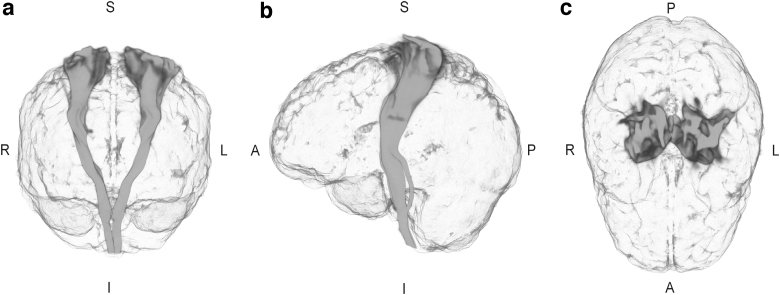
Tractography of the corticospinal tract. The tract is presented in **(a)** coronal, **(b)** sagittal, and **(c)** horizontal superior orientation. Light regions contain high and dark regions low density of streamlines. Directions: A = anterior; I = inferior; L = left; P = posterior; R = right; and S = superior.

Owing to the observed association in Global analysis, Tract-wise associations between maternal fT4 and male offspring FA were also analyzed using Model 3 covariates (Table 3). The associations were generally positive and the finding in the corticospinal tract (CST) and anterior and superior thalamic radiations and forceps minor survived FDR correction (Table 3 and Fig. 3). While associations between TSH and FA and MD in females did not survive FDR correction in Global analysis, the Tract-wise regressions in these measures are presented for interest in Supplementary Table S2.

**Table 3.** Tract-Wise Associations Between Maternal Free Thyroxine and Fractional Anisotropy in Males

	Maternal fT4 and male FA: fully adjusted model (*n* = 114)	
	β [CI]	*p*
Brainstem tracts		
Middle cerebellar peduncle	−0.029 [−0.242 to 0.183]	0.786
Medial lemniscus	0.172 [−0.037 to 0.381]	0.106
Projection fibers		
Corticospinal tract	0.305 [0.099 to 0.512]	0.004^a^
Anterior thalamic radiation	0.248 [0.054 to 0.442]	0.013^a^
Superior thalamic radiation	0.289 [0.084 to 0.493]	0.006^a^
Posterior thalamic radiation	−0.084 [−0.295 to 0.128]	0.436
Association fibers		
Superior longitudinal fasciculus	0.211 [0.011 to 0.411]	0.039
Inferior longitudinal fasciculus	0.074 [−0.138 to 0.286]	0.488
Inferior fronto-occipital fasciculus	0.156 [−0.057 to 0.369]	0.148
Uncinate fasciculus	0.216 [0.019 to 0.413]	0.032
Limbic system fibers		
Cingulate gyrus part of cingulum	0.088 [−0.115 to 0.290]	0.392
Parahippocampal part of cingulum	0.003 [−0.188 to 0.195]	0.973
Callosal fibers		
Forceps minor	0.296 [0.085 to 0.506]	0.006^a^
Forceps major	0.209 [−0.001 to 0.420]	0.051

*Post hoc* linear regressions in measures with significant findings in Global analysis. Linear regression models were used in the analyses. β represents the association between maternal fT4 and male offspring FA in each tract separately with CIs and raw *p*-values (*p*). Model is adjusted for offspring gestational week at birth, age and brain size at image acquisition, maternal age, prepregnancy BMI, socioeconomic status, cigarette smoking and alcohol use during pregnancy. Number of participants available is given in parenthesis.

^a^ *q* < 0.05.

Sensitivity analysis 1 showed (1) difference in fT4 between males with (mean = 15.033 pmol/L) and without (mean = 15.490 pmol/L) neuroimaging data (*t* = −2.303; CI = −0.850 to −0.065) but none in females and (2) no difference in brain size between males or females with and without maternal thyroid function data (Table 4). Sensitivity analysis 2 showed no change in the significance of sex-specific findings of Global analyses after adjusting Model 3 additionally with TPO antibodies. In Sensitivity analysis 3, the exclusion of mothers with overt hypothyroidism (0 participants) and hyperthyroidism (2 participants) did not change the significance of the sex-specific findings in Global analysis Model 3.

**Table 4.** Sensitivity Analysis 1: Prenatal Maternal Free Thyroxine and Offspring Brain Volume Were Compared in Those With and Without Neuroimaging and Maternal Thyroid Function Data, Respectively, in Males and Females

	No. of participants	Mean of measure
Maternal fT4 (pmol/L) in the FMC		
Males with neuroimaging data	120	15.052
Males without neuroimaging data	2960	15.492
*t*-Test, *t* [CI]^a^	−2.211 [−0.834 to −0.047]^b^	
Females with neuroimaging data	172	15.507
Females without neuroimaging data	2723	15.754
*t*-Test, *t* [CI]	−1.218 [−0.648 to 0.153]	
Brain volume (dm^3^) at 26 years		
Males with maternal thyroid data^c^	120	1.732
Males without maternal thyroid data	71	1.726
*t*-Test, *t* [CI]	0.298 [−0.034 to 0.046]	
Females with maternal thyroid data	172	1.481
Females without maternal thyroid data	88	1.443
*t*-Test, *t* [CI]	1.870 [−0.002 to 0.078]	

^a^ Student's *t*-tests were used to compare means of measures with *t*-scores and CIs.

^b^ *p* < 0.05 (uncorrected).

^c^ Maternal thyroid data includes participants with fT4 and TSH data available. Brain volume was obtained from Freesurfer estimated total intracranial volume.

FMC, Finnish Maternity Cohort.

## Discussion

In this study, we sought to elucidate the role of maternal gestational thyroid function in offspring long-term WM structural development. Our hypothesis that suboptimal maternal thyroid function is associated with altered WM microstructure in the offspring gained some support. Higher maternal fT4 was associated with stronger WM integrity, as indexed using FA, in male offspring in young adulthood. Our results suggest that maternal prenatal thyroid function, even on physiological levels, may influence offspring WM development in a sex-specific manner. To our knowledge, this is the first report of maternal prenatal thyroid function and offspring structural brain outcomes spanning adulthood.

Fetal development is fully dependent on maternal fT4 until mid-pregnancy, when parallel fetal thyroid function gradually activates.^23,39,40^ TH nuclear receptors are present already in the cerebral cortex at 9 weeks postmenstrual age (PMA) and reach 10-fold levels by 18 weeks PMA,^41^ which coincides with fetal axonal development.^42^ Compensatory mechanisms for low concentrations of THs in the fetal brain may be inadequate and neurodevelopment may be affected already at levels that are not clinically relevant for the mother.^8,43^ Furthermore, the availability of active triiodothyronine (T3) is enzymatically controlled and highly specific to brain region and PMA.^40,44^ Previous findings suggest differential effects in white versus GM, but also in more focal structures such as WM tracts.

In a recent study of a large prospective population-based cohort of 9- to 12-year-olds, the time window for the strongest association between maternal TSH and child total and cortical GM volume were reported at 8–14 weeks PMA.^24^ The group also reported an inverted U-shaped association between maternal prenatal fT4 and child total and cortical GM volume and IQ, although the associations between fT4 and brain measures diminished when adjusting for total brain volume.^24^ Maternal blood sampling in this study coincides with the time window for the strongest effect in,^24^ but contrary to their findings, but we did not observe a quadratic relation between maternal fT4 or TSH and global WM measures. This may be owing to differences in age or size of the samples, stratification by sex in our sample, or quality (volumetric vs. microstructural) and location (GM vs. WM) of brain measures. It must be noted that our microstructural measures, unlike volumetric measures, aim to only reflect local tissue properties.

Other studies of maternal thyroid function and child neuroimaging are scarce. One study in a small number of children of mothers treated for hypothyroidism during pregnancy (*n* = 22) showed smaller anterior and larger posterior area of the mid-sagittal CC, which were correlated with neuropsychological test performance.^25^ In this study, we observed an association (β = 0.296; CI = 0.085–0.506) between maternal fT4 and FA in males in the anterior CC, called *forceps minor*, that connects mostly the frontal associative regions. A trend was also observed in the posterior CC *forceps major* (β = 0.209; CI = −0.001 to 0.420). The CC is a major WM tract connecting the two cortical hemispheres, with fiber structure specific to the type of information transferred between cortical sites.^28,45^ The earlier mid-sagittal callosal observations in childhood^25^ cannot be directly compared with ours, but the findings suggest the CC might be sensitive to prenatal maternal thyroid function and further research on this major pathway is warranted.

Another study in children showed association of maternal prenatal treatment for hypothyroidism and smaller volume of the hippocampus in offspring, which correlated with memory test performance.^26^ In this study, we observed no association between the microstructure of the most prominent hippocampal connections, namely the *hippocampal part of cingulum* and the *inferior longitudinal fasciculus*, with maternal thyroid function. Our work in adults adds to the earlier morphometric findings in children, but the results are difficult to compare, and more research is needed on the role of maternal thyroid function in the development of offspring callosal and hippocampal connectivity.

Previous studies in the NFBC1986 have reported poorer scholastic performance in girls and boys^13^ and higher levels of ADHD symptoms in girls^12^ at 8 years in association with low-normal levels of maternal prenatal THs. Associations between WM structure, cognitive performance, and ADHD symptomology have been scrutinized,^46–48^ but the role of maternal TH function in these relations and in this cohort remains unexplored.

Our observations of relations between maternal fT4 and male offspring FA were generally positive and the CST, anterior and posterior thalamic radiation (ATR and PTR, respectively) and the forceps minor remained significant after FDR correction. The first half of pregnancy exhibits rapid neurogenesis and growth of axonal projections in the fetal brain. The CST is one of the first WM tracts to initiate its development and can be identified in DTI already at 13 weeks of gestation.^42^ The early axonal organization of the tract coincides with the timing of maternal blood sampling in this study, which, noting our findings, raises the possibility of influence of maternal THs in the developing male CST. The tract is also one of the first WM structures to reach a relative plateau in maturation in early 20s,^49^ supporting the relative stability of our findings at age 26 years.

Furthermore, we observed no association between maternal thyroid function and MTR in offspring's WM tracts. MTR is believed to mirror the amount of myelin sheathing the axons and has been used in the study of health and disease.^28,50,51^ In the human brain, myelination gradually begins at mid-pregnancy,^52,53^ when the fetal production of THs has already reached sufficient levels and development no longer relies on maternal supply.^21^ Reflecting this evidence, it is possible that our findings in the male CST could be rather explained by alterations in axonal than myelin properties.

The associations between maternal fT4 and offspring WM microstructure were only observed in male offspring, although a trend was present in the Global analysis of maternal TSH and MD in females (b in Table 2). WM development is known to show considerable sexual dimorphism: The volume of WM (and of GM) is larger in males already at birth^54^ and shows more rapid increase in adolescence in males compared with females,^29^ possibly relating to androgen effects.^30,31^ Maternal fT4 has been shown to associate negatively with child neurodevelopmental outcomes^40^ and birthweight,^55,56^ particularly in males.^32^ Noting this evidence and the greater vulnerability of the male fetus to environmental insults,^33^ we hypothesize that maternal prenatal thyroid function in early pregnancy modulates development of fetal axonal characteristics in the male WM. More specifically, our finding of higher FA may suggest a higher density of fibers of small diameter.^28,31^

Using cross-sectional data, we cannot, however, make inferences of causation and residual confounding cannot be excluded. The results from animal studies also do not directly translate to humans and more research elucidating the mechanisms of the effects of THs is needed.

The strengths of this study include the prospective collection of maternal blood samples and other maternal and offspring covariates. The work benefits of a relatively large sample of concordant maternal and offspring datasets, as well as the high quality of the multimodal neuroimaging data. The timing of the collection of gestational samples seems to partly coincide with the fetal development of axons^42^ but not myelin.^53^ The used measure of fT4 is considered to be available to the fetus and not bound to carrier proteins, making it suitable for our study question. Our data-driven tractography method allowed automatically extracting microstructural information of distinct WM tracts. The STROBE (strengthening the reporting of observational studies in epidemiology) guidelines were followed in this work.

Our work was limited by the lack of longitudinal data of maternal THs and offspring brain measures. Regardless of prospective birth cohort data, our study is vulnerable to selection bias and differential loss to follow-up. Interpretation of the results would have benefited of use of other biological markers in the analysis, including maternal iodine status and human chorionic gonadotropin concentration, as well as markers of child thyroid function. Of importance, our work needs replication in larger samples. Future studies should target the interactions between maternal thyroid function, stress, and inflammation as correlates of offspring neurodevelopment.^57^

## Conclusion

Maternal prenatal thyroid function was associated with offspring WM microstructure in males in early adulthood using prospective birth cohort data. The findings located most strongly to the *CST*, which early axonal development relies on maternal supply of THs. Our findings suggest that maternal thyroid function in early pregnancy may modulate offspring neurodevelopment and alter long-term axonal characteristics, but more research is needed to study the associations at different ages and brain regions and to elucidate the neurodevelopmental mechanisms in humans.

## Supplementary Material

Supplemental data

Supplemental data

Supplemental data
