# Supplementary material for: Maternal Thyroid Function During Pregnancy and Offspring White Matter Microstructure in Early Adulthood: A Prospective Birth Cohort Study
Source: Thyroid. 2023 Oct 13;33(10):1245–54. doi: 10.1089/thy.2022.0699 (PMC10611975; doi:10.1089/thy.2022.0699)
Supplement: Supplemental data [file Suppl_Data.docx]

**SUPPLEMENTAL METHODS**

**Exclusion criteria**

Exclusion criteria for the follow-up MRI include: participant was adopted, use of alcohol by the mother during pregnancy (excl. >210 ml alcohol/week), diabetes of the mother during pregnancy (onset before pregnancy, treated by insulin), premature birth (<35 weeks) and/or detached placenta, multiple births, hyperbilirubinemia requiring transfusion, type 1 diabetes, malignant tumours requiring chemotherapy, systemic rheumatologic disorders, congenital heart defects or heart surgery, epilepsy, aneurysm, bacterial infection of CNS, head trauma with loss of consciousness >30 min, brain tumour, muscular dystrophy, myotonic dystrophy, major neurodevelopmental disorders (e.g. autism), nutritional and metabolic diseases, hearing deﬁcit requiring hearing aid, vision problems (strabismus, visual deﬁcit not correctable), treatment for schizophrenia or bipolar disorder, IQ <70, low reading ability (<2 SD), special education.

**Image acquisition**

Diffusion-weighted, Magnetization transfer and T1-weighted sequences were acquired during a single session. Images were obtained on a 1.5 T scanner (Siemens, Magnetom Espree, Erlangen, Germany) using an 8-channel receiver-only head coil. T1-weighted images were acquired with a gradient recalled inversion recovery spoiled MAG prepared oversampling phase 3D sequence in sagittal slices. Parameters: TR = 2400 ms, TE = 2.56 ms, TI = 1000 ms, flip angle = 8°, matrix size = 256×256, FOV = 256×256 mm, slice thickness = 1 mm and resolution (x/y/z) = 1/1/1 mm^3^. Diffusion tensor images were acquired using a manufacturer provided diffusion-weighted spin-echo echo-planar sequence with the following parameters: TR = 9000 ms, TE = 102 ms, flip angle = 90°, matrix size = 104×104, FOV = 192×192 mm and isotropic resolution of 2.3 mm. DWI included diffusion weighted (b=1000 s/mm^2^) images in 64 non-collinear directions and one reference image. Magnetization transfer ratio was calculated from images produced with a spoiled gradient recalled 3D sequence that used magnetization transfer contrast in one of the two images. Scanning parameters were TR = 30 ms, TE = 11 ms, flip angle = 15°, matrix size = 256×192, FOV = 192×256 mm, resolution (x/y/z) = 1/1/3 mm^3^, MT pulse frequency offset = 1.5 kHz and effective flip angle = 500°.

**Tractography**

The semi-automatized AutoPTX tractography protocol utilizes FMRIB Software Library (FSL, https://fsl.fmrib.ox.ac.uk/fsl/fslwiki/FSL) tools to extract 27 white matter tracts using predefined spatial constraints in subject native space^1^. First, diffusion-weighted images were visually inspected for technical errors and anatomical variation that would impede analysis. Non-diffusion-weighted b0 images were then stripped of non-brain tissue using FSL bet^2^ with robust brain centre estimation and the resulting brain images were visually inspected. Images were corrected for coil eddy currents and susceptibility artefacts using parallelized FSL eddy_cuda8.0^3^ with parameters for outlier replacement^4^ and slice-to-volume movement correction^5^.

The resulting diffusion-weighted data were run through AutoPtx preprocessing to estimate probabilistic diffusion models in each voxel using the bedpostx algorithm. Fractional anisotropy (FA) and mean diffusivity (MD) maps were calculated, and native FA images were nonlinearly registered to the FMRIB58 high-resolution FA template, after which seed, target, stop and exclusion masks were transformed from template to native images and the output were visually inspected. In AutoPtx second stage, the results of preprocessing were given to probabilistic tractography algorithm probtrackx^6^ to trace streamlines in native diffusion space using a set of masks for each of the 27 white matter tracts. Each voxel was given a scalar value based on the number of streamlines traversing the voxel and the output was divided by the total number of streamlines of the individual. The resulting maps of normalized streamline density were thresholded by-tract^7^ and the results visually inspected for correspondence with earlier literature^8^. Tract mean values were extracted in FA and MD images using the thresholded tract binary images.

Diffusion data were available for tractography in 449 individuals, of which 1 had missing data for calculation of tensor estimate and 1 had enlarged ventricles, leaving 447 individuals for further analysis. Tracing of the right hemisphere acoustic radiation (AR) tract failed in 37/447 (8.3 %) and in the left hemisphere in 47/447 (10.5 %) and the tract was omitted from further analysis. Tracing of the medial lemniscus (ML) failed in 3 individuals, while other tracts were considered successful (Table S3). The final number of analysed tracts was 25, of which 22 were bilateral (in both hemispheres) and 3 were located between hemispheres, including the forceps minor (FMI), forceps major (FMA) and the middle cerebellar peduncle (MCP).

**Table S3. Exclusion of white matter tracts in image processing.**

| Tract | Exclusion (Original n=449) |
| --- | --- |
| Acoustic radiation^a^ | 71 (15.8 %) |
| Anterior thalamic radiation^a^ | 0 |
| Cingulate gyrus part of cingulum^a^ | 0 |
| Parahippocampal part of cingulum^a^ | 0 |
| Corticospinal tract^a^ | 0 |
| Forceps major | 0 |
| Forceps minor | 0 |
| Inferior fronto-occipital fasciculus^a^ | 0 |
| Inferior longitudinal fasciculus^a^ | 0 |
| Middle cerebellar peduncle | 0 |
| Medial lemniscus^a^ | 3 (0.7 %) |
| Posterior thalamic radiation^a^ | 0 |
| Superior longitudinal fasciculus^a^ | 0 |
| Superior thalamic radiation^a^ | 0 |
| Uncinate fasciculus^a^ | 0 |

*^a^ tract is bilateral*

**Magnetization transfer ratio**

Images with (MTon) and without (MToff) a magnetization saturation pulse were extracted of non-brain tissue using FSL bet. The MTon image was aligned with the MToff image using FSL flirt and magnetization transfer ratios (MTR) were calculated for each voxel as percent decrease in signal intensity due to transfer of magnetization:

(MToff - MTon)/MToff (S1)

To extract MTR values of WM tracts, the brain-extracted non-diffusion-weighted b0 images were linearly registered to the brain-extracted MToff images. The transforms were used to project the tractography-derived binary tract images from native diffusion space to MTR images. Transform quality was visually inspected and tract mean MTR values were extracted.

**References**

1. de Groot M, Vernooij MW, Klein S, et al. Improving alignment in Tract-based spatial statistics: Evaluation and optimization of image registration. Neuroimage 2013;76:400–411; doi: 10.1016/j.neuroimage.2013.03.015.

2. Smith SM. Fast robust automated brain extraction. Hum Brain Mapp 2002;17(3):143–155; doi: 10.1002/hbm.10062.

3. Andersson JLR, Sotiropoulos SN. An integrated approach to correction for off-resonance effects and subject movement in diffusion MR imaging. Neuroimage 2016;125:1063–1078; doi: 10.1016/j.neuroimage.2015.10.019.

4. Andersson JLR, Graham MS, Zsoldos E, et al. Incorporating outlier detection and replacement into a non-parametric framework for movement and distortion correction of diffusion MR images. Neuroimage 2016;141:556–572; doi: 10.1016/j.neuroimage.2016.06.058.

5. Andersson JLR, Graham MS, Drobnjak I, et al. Towards a comprehensive framework for movement and distortion correction of diffusion MR images: Within volume movement. Neuroimage 2017;152:450–466; doi: 10.1016/j.neuroimage.2017.02.085.

6. Hernandez-Fernandez M, Reguly I, Jbabdi S, et al. Using GPUs to accelerate computational diffusion MRI: From microstructure estimation to tractography and connectomes. Neuroimage 2019;188:598–615; doi: 10.1016/j.neuroimage.2018.12.015.

7. Groot M, Ikram MA, Akoudad S, et al. Tract‐specific white matter degeneration in aging: The Rotterdam Study. Alzheimer’s & Dementia 2015;11(3):321–330; doi: 10.1016/j.jalz.2014.06.011.

8. Wakana S, Jiang H, Nagae-Poetscher LM, et al. Fiber Tract–based Atlas of Human White Matter Anatomy. Radiology 2004;230(1):77–87; doi: 10.1148/radiol.2301021640.
