# Supplementary material for: Maternal Thyroid Function During Pregnancy and Offspring White Matter Microstructure in Early Adulthood: A Prospective Birth Cohort Study
Source: Thyroid. 2023 Oct 13;33(10):1245–54. doi: 10.1089/thy.2022.0699 (PMC10611975; doi:10.1089/thy.2022.0699)
Supplement: Supplemental data [file Suppl_TableS1.docx]

| **Supplemental Table 1. Associations between maternal thyroid hormones and offspring Global brain microstructural measures in the sample of males and females combined** | | | | | | |
| --- | --- | --- | --- | --- | --- | --- |
| **Global WM measures (n)** | **Model 1 (n= 292)** | | **Model 2 (n=279)** | | **Model 3 (n=279)** | |
| **FT4** | **β (95% CI)** | **p** | **β (95% CI)** | **p** | **β (95% CI)** | **p** |
| FA | 0.054 (-0.012, 0.120) | 0.107 | 0.051 (-0.015, 0.118) | 0.130 | 0.056 (-0.009, 0.122) | 0.094 |
| MD | -0.061 (-0.128, 0.005) | 0.071 | -0.061 (-0.128, 0.005) | 0.072 | -0.059 (-0.126, 0.007) | 0.080 |
| MTR | -0.010 (-0.086, 0.066) | 0.799 | -0.006 (-0.084, 0.071) | 0.873 | 0.002 (-0.074, 0.078) | 0.959 |
| **TSH** |  |  |  |  |  |  |
| FA | 0.044 (-0.022, 0.110) | 0.189 | 0.043 (-0.024, 0.109) | 0.211 | 0.036 (-0.030, 0.101) | 0.292 |
| MD | -0.028 (-0.095, 0.039) | 0.410 | -0.036 (-0.103, 0.031) | 0.292 | -0.039 (-0.106, 0.028) | 0.251 |
| MTR | 0.056 (-0.020, 0.132) | 0.151 | 0.056 (-0.021, 0.133) | 0.157 | 0.050 (-0.026, 0.126) | 0.196 |
| *Linear mixed-effects models were used in the analysis.* *β represents the association between thyroxine (FT4) or thyroid stimulating hormone (TSH) and neuroimaging measures fractional anisotropy (FA), mean diffusivity (MD) and magnetisation transfer ratio (MTR) in all 14 white matter tracts with 95% confidence intervals (95% CI) and raw p-values (p). Model 1 is adjusted for offspring sex and age at image acquisition, Model 2 additionally for maternal age, prepregnancy body mass index (BMI), socioeconomic status, cigarette smoking and alcohol use during pregnancy, and Model 3 additionally for offspring full gestational weeks at birth and brain size at early adulthood image acquisition. Number of participants available for each model is given in parenthesis.* | | | | | | |
