# Supplementary material for: Maternal Thyroid Function During Pregnancy and Offspring White Matter Microstructure in Early Adulthood: A Prospective Birth Cohort Study
Source: Thyroid. 2023 Oct 13;33(10):1245–54. doi: 10.1089/thy.2022.0699 (PMC10611975; doi:10.1089/thy.2022.0699)
Supplement: Supplemental data [file Suppl_TableS2.docx]

|  | **Female FA: Fully adjusted model (n=172)** | | **Female MD: Fully adjusted model (n=172)** | | |
| --- | --- | --- | --- | --- | --- |
|  | **β (95% CI)** | **p** | **β (95% CI)** | **p** |  |
| **Brainstem tracts** |  |  |  |  |  |
| Middle cerebellar peduncle | 0.098 (-0.070, 0.266) | 0.251 | -0.041 (-0.209, 0.127) | 0.631 |  |
| Medial lemniscus | 0.100 (-0.066, 0.267) | 0.237 | 0.066 (-0.101, 0.233) | 0.437 |  |
| **Projection fibres** |  |  |  |  |  |
| Corticospinal tract | 0.140 (-0.023, 0.303) | 0.092 | -0.096 (-0.263, 0.071) | 0.258 |  |
| Anterior thalamic radiation | 0.159 (-0.003, 0.320) | 0.054 | -0.166 (-0.328, -0.004) | 0.044 |  |
| Superior thalamic radiation | 0.233 (0.071, 0.395) | 0.005^a^ | -0.190 (-0.356, -0.024) | 0.025 |  |
| Posterior thalamic radiation | 0.037 (-0.126, 0.199) | 0.656 | -0.056 (-0.219, 0.107) | 0.495 |  |
| **Association fibres** |  |  |  |  |  |
| Superior longitudinal fasciculus | 0.181 (0.017, 0.345) | 0.031 | -0.241 (-0.399, -0.084) | 0.003^a^ |  |
| Inferior longitudinal fasciculus | 0.086 (-0.078, 0.251) | 0.302 | -0.156 (-0.321, 0.009) | 0.064 |  |
| Inferior fronto-occipital fasciculus | 0.124 (-0.044, 0.292) | 0.146 | -0.153 (-0.316, 0.010) | 0.066 |  |
| Uncinate fasciculus | 0.092 (-0.066, 0.249) | 0.252 | -0.137 (-0.297, 0.023) | 0.092 |  |
| **Limbic system fibres** |  |  |  |  |  |
| Cingulate gyrus part of cingulum | 0.093 (-0.075, 0.261) | 0.275 | -0.154 (-0.316, 0.008) | 0.062 |  |
| Parahippocampal part of cingulum | 0.230 (0.077, 0.383) | 0.003^a^ | -0.010 (-0.179, 0.159) | 0.907 |  |
| **Callosal fibres** |  |  |  |  |  |
| Forceps minor | 0.129 (-0.039, 0.296) | 0.131 | -0.260 (-0.424, -0.097) | 0.002^a^ |  |
| Forceps major | 0.008 (-0.156, 0.172) | 0.920 | 0.025 (-0.139, 0.190) | 0.762 |  |

**Supplemental Table 2. Tract-wise regressions between maternal thyroid stimulating hormone (TSH) and fractional anisotropy (FA) and mean diffusivity (MD) in females**

*Linear regression models were used in the analyses. β represents the association between maternal free thyroxine (FT4) and male offspring fractional anisotropy (FA) in each tract separately with 95% confidence intervals (95% CI) and raw p-values (p). Models are adjusted for offspring gestational week at birth, age and brain size at image acquisition, maternal age, prepregnancy BMI, socioeconomic status, cigarette smoking and alcohol use during pregnancy. Number of participants available is given in parenthesis. ^a^ q < 0.05.*
